# Developing a psychoeducational programme for caregivers of people with intellectual disability

**DOI:** 10.4102/ajod.v12i0.1195

**Published:** 2023-09-22

**Authors:** Bonita K. Gordon, Nontembeko J. Bila

**Affiliations:** 1 Department of Social Work and Criminology, Faculty of Humanities, University of Pretoria, Pretoria, South Africa

**Keywords:** intellectual disability, formal paid caregivers, informal unpaid caregivers, psychoeducational programme, caregiver distress

## Abstract

**Background:**

In the Western Cape, South Africa, a significant number of individuals with intellectual disabilities are cared for by caregivers who receive little or no compensation, education or support. Despite the unique challenges faced by these caregivers, no psychoeducational programmes have been implemented for this particular population.

**Objectives:**

The study aimed to examine the factors contributing to caregiver distress and develop a solution in the form of a psychoeducational programme for caregivers.

**Methods:**

A mixed-methods research approach was employed. The qualitative phase involved exploratory research to gather fundamental information and gain new insights into caregiver distress. The quantitative phase utilised a ‘one-group pre-test, post-test design’ with a Likert-scale questionnaire to enable meaningful interpretations and comparisons of the psychoeducational programme’s impact and value. The paired t-test was employed to determine significant differences between pre-test and post-test results.

**Results:**

The statistical findings demonstrated a significant increase in knowledge, with 99% of respondents indicating a positive impact in reducing caregiver distress and 85% feeling better equipped to care for individuals with intellectual disabilities.

**Conclusion:**

The psychoeducational programme developed in this study had a positive effect on reducing caregiver distress.

**Contribution:**

This knowledge provides valuable insights for healthcare professionals in designing relevant intervention programmes, offering support and providing resources not only for individuals with intellectual disabilities but also for their caregivers.

## Introduction

Extensive research has demonstrated the demanding and burdensome nature of caring for individuals with intellectual disability (ID) (Ezeonu et al. 2021; McConnell & Savage 2015; Panicker & Ramesh 2019; Steiner 2011; Ugwuanyi et al. 2022). In the Western Cape region, the responsibility of caring for 98% of individuals with ID falls upon their caregivers, who often remain as unacknowledged brave individuals within the ID community (Kench 2016; McKenzie, McConkey & Adnams 2016). These caregivers frequently sacrifice their aspirations and receive little to no compensation or recognition (Coetzee 2016; Kench 2016; Perkins 2009). Emotionally and physically neglected, they are at risk of burnout and depression (Coetzee 2016).

As individuals with ID rely on their caregivers’ judgement in decision-making, the burden of care falls heavily on these caregivers. Their responsibilities encompass managing challenging behaviours, navigating the healthcare, legal and social systems, as well as attending to daily living activities such as grooming (Vecchio, Cybinski & Stevens 2009). The lack of evidence-based programmes to support caregivers has left them emotionally depleted (Smith Da Walt, Greenberg & Mailick 2018). Targeted psychoeducational programmes have proven to be effective in reducing caregiver stress and improving outcomes for both caregivers and individuals with ID (Smith Da Walt et al. 2018).

While numerous studies have explored caregiving and ID (Adnams & Johns 2016; Lauderdale-Littin & Blacher 2017; McKenzie et al. 2016; Smith Da Walt et al. 2018; Yoong & Koritsas 2012), as well as the prevalence of ID in the Western Cape (Kleintjes et al. 2006; McKenzie et al. 2016), and ID resulting from foetal alcohol syndrome and its prevalence in the high-risk areas of the Western Cape (May et al. 2000, 2013), there is a lack of literature on a targeted psychoeducational programme specifically designed for both formal (paid) and informal (unpaid) caregivers of individuals with ID in the Western Cape. The unique challenges faced by this population necessitate attention, and this study aims to address this research lacuna.

This article aims to demonstrate that a targeted psychoeducational programme incorporating: (1) disability education, (2) problem-solving guidelines and (3) opportunities for social support among individuals with shared experiences can effectively alleviate caregiver distress. The theoretical foundations of the present study are the biopsychosocial theoretical framework and the strength-based approach. The biopsychosocial model, as distinguished by Engel (1977, 2003), positions caregivers as experts who contribute to problem-solving solutions. This model views disability as a complex interplay of three factors: physical (bio), psychological (behavioural) and social (cultural environment) (Petasis 2019). By adopting the biopsychosocial model, this study gained a comprehensive framework for understanding the needs, aspirations and interactions of individuals with ID and their caregiver families (McDaniel & Pisani 2012). Integrated with the biopsychosocial model, the strength-based approach was employed as a foundational principle for the study. The strength-based approach posits that individuals facing adversity and crises develop resilience and resourcefulness. They acquire skills to overcome challenges and view these obstacles as opportunities to showcase their resourcefulness and respond in culturally meaningful ways (Moorkath, Ragesh & Hamza 2019).

## Research methods and designs

### Research approach and design

The study comprised two distinct phases: (1) a qualitative phase followed by (2) a quantitative phase. In the initial qualitative phase, the primary objectives were to delve into the experiences of caregivers supporting individuals with IDs and identify their specific knowledge and skill requirements. This phase aimed to gain insights into the challenges faced by caregivers and their unique needs. Upon the commencement of the second phase, the focus shifted towards designing a psychoeducational programme tailored to caregivers of individuals with IDs, drawing upon the needs and insights identified in the qualitative phase. The key goal was to enhance caregivers’ knowledge of ID and equip them with the necessary skills to provide effective care. To accomplish this, several steps were taken in the quantitative phase. Firstly, a pre-test assessment was conducted to evaluate caregivers’ existing knowledge of caregiving for individuals with IDs. This provided a baseline measure against which the effectiveness of the psychoeducational programme could be assessed. Secondly, the psychoeducational programme was implemented through comprehensive training sessions provided to the caregivers. These sessions aimed to address the identified knowledge and skill gaps and empower caregivers with the necessary tools to meet the challenges associated with IDs. Following the completion of the training, a post-test assessment was conducted to gauge the caregivers’ knowledge of caregiving for individuals with IDs. By comparing the pre-test and post-test results, the effectiveness of the programme was analysed, allowing for an evaluation of its impact on caregivers’ knowledge and skills. Based on the research findings from both phases, conclusions were drawn, and recommendations were made for further refinement and implementation of the developed psychoeducational programme. These insights served as a valuable guide for future enhancements and optimisations, ensuring that the programme could effectively support caregivers in their vital role of caring for individuals with IDs.

The study employed an embedded mixed-methods design, which is a variant of the broader mixed-methods research approach (Ivankova, Creswell & Plano Clark 2011). In this design, one data set plays a supportive, secondary role, while the study primarily relies on the other data set. The two data sets were collected simultaneously, allowing for a comprehensive analysis of the research question (Creswell & Clark 2011; Delport & Fouchè 2011). The decision to utilise the embedded mixed-methods design was driven by the need to investigate multiple groups within a specific time, primarily employing quantitative data collection methods. This design was deemed appropriate as it allowed for close integration of data collection and analysis at various stages, as highlighted by Creswell and Clark (2011) and Fetters, Curry and Creswell (2013). By adopting the embedded mixed-methods design, the study was able to capitalise on the strengths of both quantitative and qualitative data, providing a more comprehensive understanding of the research topic. This design facilitated a nuanced exploration of the research question and enriched the overall analysis by incorporating different perspectives and insights derived from the two data sets.

### Research setting and population

The research population consisted of both informal and formal caregivers responsible for individuals with ID in the Western Cape, South Africa. Informal caregivers encompassed unpaid individuals who were primarily biological family members, including mothers, fathers, siblings, grandparents, as well as other relatives, foster parents and community members directly involved in caregiving for a person with ID. On the other hand, formal caregivers included paid professionals such as care assistants, nurses and housemothers working in residential homes, group homes and education centres specifically catering to individuals with ID in the Western Cape. Additionally, supervisors in protective workshops for individuals with ID were also considered as formal paid caregivers. It is important to observe that the study excluded nurses who provided care for individuals with ID in larger institutional settings such as hospitals. The focus of the study primarily centred on informal and formal caregivers directly involved in the day-to-day caregiving of individuals with ID in various community-based and residential settings in the Western Cape.

### Study selection and inclusion criteria: Qualitative phase (informal caregivers)

In the qualitative approach, non-probability sampling was employed because of the lack of precise information regarding the population size of informal caregivers in the Western Cape, resulting in an unequal chance of selection (Creswell & Clark 2011; Maree & Pietersen 2011a; Strydom 2011). Purposive sampling was utilised, wherein the authors’ judgement guided the selection of participants for the study. The sample consisted of caregivers who met the predefined criteria for the target population (Maree & Pietersen 2011a; Strydom 2011). The selection criteria required participants to be full-time caregivers for individuals with ID and residents of the Western Cape. A sample size of 25 informal caregivers was deemed sufficient to fulfil the objectives of the qualitative phase of the study.

#### Participants

Out of the 25 selected participants, 21 were females, accounting for 57% of the sample, with most of them being lone caregivers. Among the participants, there were four males. The predominant role among the caregivers was held by mothers, totalling 15 participants. Additionally, the sample included four fathers and one sister who were actively involved in caregiving responsibilities. Interestingly, five participants had no biological relation to the individuals with an ID they were caring for. Instead, they were providing care through foster arrangements or private agreements. One noteworthy case was a neighbour who stepped in to care for a person with an ID after recognising that the biological mother was struggling to cope with the responsibilities. The ages of the participants ranged from 32 to 70 years, representing a diverse age range within the caregiver sample.

Most informal caregivers in the study were responsible for the care of a single individual with ID. However, there were a few notable cases: one caregiver was caring for two siblings with ID, and another caregiver had the responsibility of caring for three biological children diagnosed with ID.

In four instances, both the father and mother participated in the interview as caregivers for the same individual with an ID. This suggests a shared caregiving role and involvement of both parents in the care process. Regarding the gender profile of the individuals with ID, it was observed that the majority were males, totalling 18, while 6 were females. Among the individuals with ID, 10 were minors, meaning they were under 18 years, and 14 were adults. The ages of individuals with ID ranged from 4 to 40 years. It is worth noting that in all cases, the individuals with ID resided at home with their respective caregivers, indicating a familial caregiving arrangement.

### Study selection and inclusion criteria: Quantitative phase (formal caregivers)

In the quantitative phase of the study, probability sampling was employed as the authors had access to a list of names, allowing for a completely random selection process. To obtain this list of names, invitations were sent out to various group homes, workshops and day centres. Caregivers who received the invitation were encouraged to respond by completing a registration form provided via email. As a result, the authors compiled a list of formal caregivers who had completed the registration form, creating a pool from which a random selection could be made. This random selection process adhered to the principles of probability sampling (Maree & Pietersen 2011a; Strydom 2011). The selection criteria for the employed caregivers in the quantitative phase were as follows: (1) they had to be formally employed by an organisation, such as a residential group home or protective workshop; (2) the primary focus of their employment had to involve taking care of a person with ID and (3) they needed to reside in the Western Cape. These criteria ensured that the sample of formal caregivers represented those who were employed within relevant caregiving contexts in the Western Cape.

To ensure a comprehensive representation of the study population, the largest feasible sample size was obtained (Strydom 2011). This approach aimed to include participants who encompassed various socioeconomic classes and genders, thereby enhancing the generalisability of the findings to the entire population (Strydom 2011). To meet the objectives of the quantitative phase, a predetermined sample size of 100 respondents was selected to participate in the training (Maree & Pietersen 2011a). This sample size was deemed sufficient to achieve the desired outcomes of the study and to provide robust data for analysis and interpretation.

#### Respondents

The research training predominantly attracted female caregivers, accounting for 94% of the participants, while only 6% were male caregivers. Among the participants, 47% had acquired their caregiving knowledge through workplace experience without formal caregiver training. Furthermore, they had received no specific training related to ID. On the other hand, 49% of the respondents reported having a formal qualification or training as a caregiver. However, it should be noticed that 26% of those individuals had not received training specifically focused on ID. As a result, a significant proportion of the respondents, approximately 73%, lacked training in the context of ID. In terms of income, 57% of the participants reported earning less than R5000.00 per month.

### Data collection

During the qualitative phase, a semi-structured interview schedule was developed to collect data, aiming to obtain a comprehensive understanding of the experiences and needs of unpaid caregivers of individuals living with ID (Greeff 2011). To enhance the transferability of the findings, experts in the field were also interviewed alongside informal caregivers, as gathering information from multiple sources is considered valuable (Anney 2014; Schurink, Fouché & De Vos 2011).

The experts who participated in the study were healthcare and educational professionals specialising in ID within the Western Cape. Their expertise was crucial in providing valuable insights, guidance and feedback on the content of the psychoeducational programme and questionnaires. Additionally, a qualified statistician reviewed the questionnaires to ensure their validity and reliability. The professionals’ role as experts allowed them to anticipate and identify potential challenges or omissions that could hinder the study’s objectives. Their input was instrumental in shaping the programme and ensuring its effectiveness. On the other hand, the interviews conducted with informal caregivers aimed to capture their lived experiences and understand their specific needs as caregivers of individuals with ID. Their perspectives and first-hand accounts provided valuable insights into the challenges, concerns and support requirements faced in their caregiving roles.

The development of the psychoeducational programme was a meticulous process that incorporated a thorough review of existing literature and the insights gained from the first qualitative phase of the study. The identification of various needs and skills required by caregivers of individuals with ID served as a foundation for designing the programme. To ensure a comprehensive and effective programme, a collaborative approach was adopted, involving both experts and caregivers. Their collective expertise and experiences, combined with the existing literature, contributed to the development of a well-rounded training manual. The training manual was carefully crafted, considering the unique challenges faced by caregivers and drawing upon evidence-based practices. It aimed to address the identified needs, provide valuable education on ID, offer practical problem-solving guidelines and foster opportunities for social support. The manual served as a guide for the implementation of the psychoeducational programme, providing structured training sessions that focused on empowering caregivers with the necessary knowledge and skills to enhance their caregiving experiences and support the well-being of individuals with ID.

The psychoeducational programme comprised the following four modules: (1) psychoeducation on intellectual disability for caregivers; (2) psychosocial impact on caregivers; (3) strengthening the family unit and (4) accessing and developing resources.

### Module A: Psychoeducation on intellectual disability for caregivers

Module A aimed to provide caregivers with essential knowledge on ID, addressing misconceptions and promoting accurate understanding. It focused on identifying and addressing cognitive distortions that could hinder effective caregiving. The module also emphasised meeting the care needs of individuals with ID, covering topics such as personal care, communication strategies, independence promotion and the importance of medication adherence.

### Module B: Psychosocial impact on caregivers

Module B addressed the psychosocial challenges faced by caregivers and provided coping strategies and support. It explored caregivers’ daily challenges and offered insights into effective coping mechanisms. The module introduced the concept of resilience and provided strategies for developing resilience in the caregiving role. Emphasising the importance of self-care, practical exercises were introduced to promote self-care practices. Caregivers learned about emotional acceptance and commitment as crucial aspects of their well-being. They were encouraged to strive for being a ‘good enough’ caregiver, recognising that perfection is not the goal. The module emphasised the significance of having an internal locus of control and included a practical exercise on understanding the dichotomy of control.

### Module C: Strengthening the family unit

Module C focused on empowering caregivers to address challenging behaviours exhibited by individuals with ID, strengthening the family unit in the process. Caregivers gained insights into the causes of these behaviours, learning to identify triggers and understand underlying factors. Proactive approaches and effective techniques were provided for managing challenging behaviours. Additionally, the module covered managing family conflict and promoting household safety.

### Module D: Accessing and developing resources

Module D focused on equipping caregivers with the skills of resourcefulness, addressing the frustrations expressed by caregivers regarding the lack of resources for individuals with ID. The module aimed to empower caregivers in identifying and accessing resources while being assertive and proactive in meeting their needs. Caregivers learned techniques for identifying available resources within their community or support networks. They were encouraged to maintain thorough records of resources, ensuring easy access when needed. The module also aimed to enhance caregivers’ mindset towards developing their resources, such as building a support team equipped with the necessary skills.

The training event focused on formal caregivers and served the research purpose. To collect data, pre- and post-test questionnaires were administered. The pre-test questionnaire was given before the intervention programme to gather baseline information. The Likert-scale questionnaire used in the survey was developed based on a comprehensive study of caregiver issues (Browne & Greene 2005). Following the completion of the intervention programme, the post-test questionnaire was administered to assess the increase in knowledge among participants and their perception of the programme’s positive impact on alleviating caregiver distress. The pre- and post-test results were combined and analysed, allowing the researcher to draw valuable interpretations and make comparisons regarding the effectiveness and value of the psychoeducational programme (Fouché, Delport & De Vos 2011; Pietersen & Maree 2011). To ensure the reliability of the data, an independent coder was employed. Their involvement added to the trustworthiness of the findings.

### Data analysis

During the qualitative phase, the interviews were transcribed from audio recordings into written text. Any interviews conducted in Afrikaans were translated into English. The transcribed data were organised and stored in an electronic folder for easy access during data reduction, representation and interpretation (Nieuwenhuis 2011). To gain a deeper understanding of the non-textual data, the audio recordings were played and reviewed multiple times (Schurink et al. 2011). Thematic analysis was employed to identify emerging themes, patterns and their underlying meanings (Roberts, Dowell & Nie 2019). This analytical approach aimed to uncover and critically assess the recurring themes present in the interview data. To ensure the credibility of the analysis, the emerging understanding was tested and evaluated, considering aspects that may not have been explicitly stated in the data but could be significant for analysis. The absence of new themes indicated that data saturation had been reached, indicating sufficient depth and coverage of the information (Schurink et al. 2011). The psychoeducational programme developed in the second phase for formal caregivers was based on valuable information obtained from literature reviews and the empirical findings of the in-depth interviews with informal caregivers. This ensured that the content of the programme was grounded in both theoretical knowledge and the practical experiences of caregivers.

During the second phase (quantitative), the data analysis was conducted in collaboration with a statistician. To ensure consistency and accuracy, a codebook and a memorandum were developed outlining the scoring criteria for the responses. The data were coded by assigning numerical values to the responses provided by the participants who completed the pre- and post-test questionnaires. Each participant’s score was calculated by summing the assigned values for their responses. The scoring system involved categorising attitudes on a scale of 1–4, with 1 representing ‘strongly agree’ and 4 representing ‘strongly disagree’. The values for each participant’s responses were then added to determine their overall attitudes or values. This scoring method, as guided by Delport and Roestenburg (2011) and Maree and Pietersen (2011b), allows for quantitative analysis of the data, enabling comparisons and statistical interpretations to be made. The collaboration with a statistician ensured that the data analysis process was rigorous and accurate.

The data were prepared for analysis using Microsoft Excel 365, as recommended by Fouchè and Bartley (2011). The pre- and post-test responses were scored and merged to facilitate comparison and analysis. This scoring method enabled the examination of changes in knowledge levels between the pre- and post-test sections of the preliminary intervention programme. By comparing the scores, it was possible to evaluate the participants’ knowledge before the intervention and assess the knowledge gained during the programme. To present the descriptive results clearly and understandably, charts and tables were utilised. Visual representations, such as figures and graphs, are effective tools for conveying information and enhancing comprehension, as suggested by Creswell and Clark (2011).

Moreover, to assess the strength of the comparison for each question, a paired t-test was employed. This statistical test involved calculating the numerical difference between the post-responses and the pre-responses for each participant and each question. These average differences were then evaluated using a t-distribution, as they follow a t-distribution pattern. The purpose of the t-test was to determine whether the differences observed were significantly greater than zero, indicating a meaningful change. Because we were comparing two responses from the same participants, this test is referred to as a ‘paired t-test’, as described by Pietersen and Maree (2011).

### Ethical considerations

The study obtained ethical approval from the Human Research Ethics Committee of the University of Pretoria (HREC reference no.: HUM034/0720). Informed consent was obtained from all participants, ensuring that they were fully aware of the purpose, procedures and potential risks involved in the study. Ethical principles such as avoiding harm and deception, maintaining confidentiality and providing debriefing to participants were strictly adhered to throughout the research process. Pseudonyms were used to ensure the confidentiality of the participants.

## Results

### Qualitative research findings: First phase

The thematic analysis led to the emergence of the following main categories of themes.

### The lack of psychoeducation of the caregiver

The findings highlight that a lack of psychoeducation among caregivers contributes to caregiver distress. The evidence revealed that some participants held misconceptions, such as believing they were personally responsible for the disability or that their family was cursed. Many participants expressed dissatisfaction with the limited information provided by professionals in the field, prompting them to conduct their research. The following verbatim statements from participants support these assertions:

‘I was on antibiotics, and I did not know that it would interfere with my pregnancy. I always told my husband that I am the cause for my child being the way he is.’ (Married mother, 21-year-old son with ID)

‘Look, I did not know much at the time. I had to figure things out for myself as life went on. Because I did not know what it [*intellectual disability*] was, I had to investigate it, read about it, go to workshops, go to Red Cross, and educate myself and, you know, all the mothers sit and talk to each other and give advice. I was not clued up.’ (Divorced mother, 31-year-old son with ID)

‘People were telling me that it’s witchcraft and that someone is jealous of me.’ (Married mother, three adult children with ID)

Most caregivers expressed the difficulties they face in coping with the challenging behaviour of individuals with ID, often feeling uncertain about how to effectively manage such behaviour. Some caregivers candidly admitted to resorting to desperate measures in these situations. For instance, one mother shared her regret about a past incident where she resorted to physically hitting her child, resulting in the child requiring hospitalisation. Another mother mentioned using cigarettes to calm down her son, and disturbingly, she even mentioned the possibility of using extreme measures such as burning him with a kettle if the violence were to escalate:

‘The cigarettes calm him down a bit and I know that he needs them; so, I had to make a plan [*during COVID-19*] so that he could have a cigarette … he used to choke me and he used to break a lot of things. There were times I would switch on the kettle because I would think if this child did any harm, I would burn him. That is the desperation.’ (Single mother, 21-year-old son with ID)

*‘Hy het my hele huis opgebreek. Toe kon ek nie anders nie en toe slaan ek hom, en toe slaan ek hom hospital toe. Ek het myself verwyt dat ek hom geslaan het.’* [*He damaged my home. I felt that I had no choice and so I hit him. I beat him to the point he had to go to hospital. I blamed myself for hitting him like that.*]

*‘n Mens weet nie hoe om na ‘n gestremde kind te kyk nie. Sy slaan my so dat ek sterretjies sien. Ek het nie geweet wat om te maak nie.’* [*One does not know how to take care of someone with a disability. She hit me to the extent that I saw stars. I didn’t know what to do.*] (Pensioner, 28-year-old daughter with ID)

### Socioeconomic impact of caregiving

The findings of the study reveal that the caregiver role significantly affected the participants’ ability to work and support themselves and their families. Many caregivers relied solely on the disability grant as their primary source of income:

‘Lisa is very expensive; she eats the whole day. Lisa’s disability grant is my only income. I used to iron but I can’t even go char because my sister refuses to watch her for me because of her behaviour. I must pay rent with her money.’ (Single mother, 17-year-old daughter with ID)

Consequently, many caregivers expressed anxiety about the future care and provision for the person with ID when they were no longer able to fulfil the caregiving role:

‘I always have this fear and anxiety. Who will take care of him when we are not there anymore. No one understands him as we do.’ (Married mother, 22-year-old son with ID)

‘I say “God, if you have to take me then please take Adrian with me so that I can go in peace.”’ (Pensioner, 37-year-old son with ID)

### Caregiver resource constraints

The evidence revealed that many participants attempted to seek assistance but encountered significant challenges. They consistently found that medical, police and social work services were either unavailable or inadequately equipped to support their needs:

‘The system fails us. The social workers were always unavailable. She never pitched up and was never even an apology. No professional courtesy. I mean that is their job. She has taken a fat cat salary; the children are so vulnerable and falling through the cracks and we can’t get help for them.’ (Single mother, two teenage children with ID)

‘I used to call the police and they just came and couldn’t do much. The police stopped coming when I call because they don’t know how to deal with this. He started to get aggressive and attacked us physically. We went to the Voice for help. The Voice is the newspaper, we were on the front of the Voice, I appealed to the public to help us, but it didn’t help.’ (Widow, 31-year-old son with ID)

### Psychosocial impact on the caregiver

The evidence indicates that caregiving for a person with ID has a profound psychosocial impact on the caregivers. The participating caregivers expressed their individual experiences of lacking support and feeling isolated. Additionally, they faced community stigmatisation, which further contributed to their sense of isolation. Family relationships were also affected by the demands of caregiving, and caregivers struggled to prioritise their own mental and physical well-being:

‘I am not happy about the breakup with the father. I am very sad about that. I don’t have friends since 2017. Since he is born.’ (Single mother, 4-year-old son with ID)

The evidence reveals that a significant number of participants shared their experiences of stigmatisation, which left them feeling vulnerable and at risk. They recounted instances of being subjected to mockery and hurtful name-calling by members of their community, directed towards both themselves as caregivers and the individuals with ID under their care:

‘I walk with my kids in the mall. In public people mock and point at my kids. I tell myself to stay strong. I have realised society has cast them out. They call us the “house of stupid people” and they labelled my house. People can call you names, and it can stick like glue!’ (Married mother, three adult children with ID)

The present research also uncovered that some participants found their role as caregivers to be rewarding and fulfilling. The study delved into the factors that contributed to their positive experiences. Here are a few direct quotes from the participants themselves:

‘They have a mind of their own and we need to be patient with them. We need to be accepting of people that are different. What do we call different? There is nothing wrong with them. They are God’s people as they are your way to Heaven. They are special people.’ (Single mother, 13-year-old son with ID)

### Quantitative research findings: Second phase

#### Pre-test versus post-test results

The caregivers were asked to rate their confidence in their knowledge of the following topic as presented in Table 1.

**TABLE 1:** Statistical summary of the pre-versus post-test results.

Topics	Knowledge of the topic before training (%)	Knowledge of the topic after training (%)
Understanding intellectual disability	36	92
Developing resilience	27	90
Resourcefulness	22	90
Managing behaviour that challenges	27	89
Emotional acceptance	28	88
Cognitive distortions	24	89
Understanding the dichotomy of control	21	89
Self-care and coping strategies	27	88
The empowered family	23	89

Note: The intervention of the psychoeducational programme was applied after the pre-test and before the post-test. A significant number of respondents moved from a negative response in the pre-test to a positive response in the post-test. It can be concluded that the intervention of the psychoeducational programme was the reason for the positive responses.

The results revealed that an overwhelming majority of the respondents, specifically 99%, expressed that the psychoeducational programme had a positive impact in alleviating caregiver distress. As part of the data collection process, respondents were asked to provide additional comments to elaborate on their answers. Here are some verbatim excerpts from their written responses:

Would this psychoeducational programme have a positive impact on alleviating caregiver distress?:

- ‘I learned I am not a failure.’
- ‘Meaningful to know what to do.’
- ‘It provided an opportunity to reflect on myself and how I have been doing my job.’
- ‘This program gave me clarity on some situations.’
- ‘It helps me to make sound decisions on behalf of my client.’

In the following section, the respondents’ views on whether they felt more equipped to take care of a person with ID after having attended the training were explored.

**FIGURE 1:**
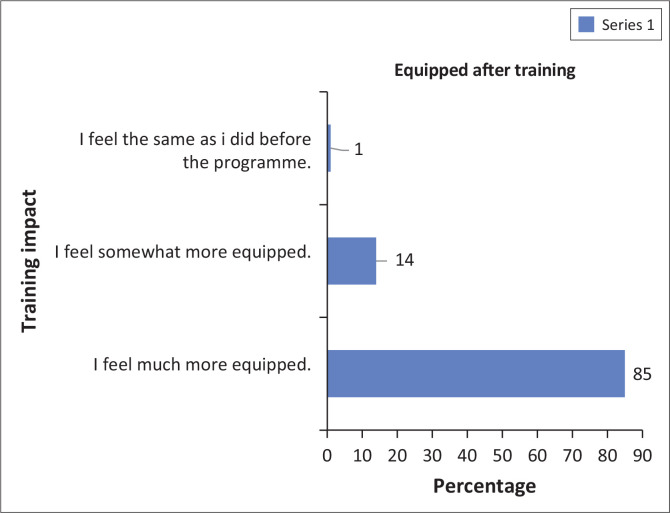
Degree of feeling equipped after training.

Based on Figure 1, the data indicate that 85% of the respondents felt much better equipped to take care of a person with ID after completing the training. An additional 14% felt somewhat more equipped, while only 1% reported feeling the same as before the training.

The respondents expressed that the psychoeducational programme significantly increased their confidence and competence as caregivers. They highlighted that the programme improved their understanding of ID, which directly enhanced their ability to provide effective care. The following are verbatim responses that support this:

- ‘I learned stuff I never knew about people with intellectual disability.’
- ‘I have gained so much knowledge with this program.’
- ‘I learned a lot more about what the caregiver must do.’
- ‘The material is designed to equip the caregiver, but also equip the person with ID to function better.’

## Discussion

A caregiver is any individual who provides nurturing acts or attends to the needs of someone requiring such assistance. Caregiving involves a range of responsibilities, including offering emotional support, aiding with healthcare and medical requirements, assisting with daily activities and facilitating referrals to appropriate medical professionals when necessary (Schulz & Edin 2016). Caregivers can be categorised as either formal or informal. The distinction between these two categories lies in the caregiver’s level of expertise and knowledge, as well as the compensation they receive for their caregiving services (Musich et al. 2018). Formal caregivers are recognised as having the necessary skills and knowledge to provide care to recipients.

The primary objective of the study was to investigate the underlying factors that contribute to caregiver distress and subsequently develop a viable solution in the form of a psychoeducational programme specifically designed for caregivers.

The findings of this study align with the research conducted by Ezeonu et al. (2021), which emphasises that most caregivers lack an understanding of the causes of ID. As a result, caregivers tend to attribute distinct reasons to ID, such as viewing it as a punishment from God, superstition and bewitchment, or perceiving it as a spiritual attack or demon possession (Ezeonu et al. 2021; Mkabile et al. 2021). Caregivers need to have a clear understanding of ID as it directly contributes to their ability to effectively cope with the challenges associated with caring for individuals with ID (Ezeonu et al. 2021). In a study conducted by Simpson et al. (2022), it was noticed that caregivers expressed an ardent desire for knowledge and training in this area. The research findings of Simpson et al. (2022) further indicate that the training priorities identified by caregivers include the management of problematic behaviour, knowledge and understanding of ID, and practical interventions that encompass positive support in caregiving. These studies highlight the importance of providing caregivers with education and training to enhance their understanding of ID and equip them with effective strategies for caregiving. The findings support the need for interventions that address caregivers’ knowledge gaps and prioritise areas such as behaviour management and practical caregiving approaches. By addressing these priorities, caregivers can enhance their ability to provide optimal care and support for individuals with ID.

Furthermore, this study provides unmistakable evidence that many participants lacked the necessary knowledge and training to effectively manage challenging behaviour in individuals with ID (Tilley, Ledger & Bardsley 2015). The experiences shared by the participants regarding the lack of psychoeducation align with a study conducted on caregivers of individuals with ID in Khayelitsha, a district in Cape Town, by Mkabile and Swartz (2020). These authors found that most caregivers received inadequate information from medical practitioners regarding their children’s condition.

Newcomb and Hagopian (2018) state that children with ID exhibit challenging behaviour at higher rates compared with their typically developing peers. Behaviours such as self-injury (e.g. head banging), aggression, pica (ingesting non-food items), disruption and wandering can significantly impact the quality of life for both the individual with ID and their family. These findings highlight the importance of addressing and effectively managing challenging behaviours to improve the overall well-being of individuals with ID and their families (Newcomb & Hagopian 2018).

The findings of this study align with existing literature regarding the socioeconomic impact of caregiving. Caregivers bear significant daily care responsibilities, and the sacrifices they make often have financial implications (Marsack-Topolewski 2021; Perkins 2009; Marsack-Topolewski & Church 2019). The findings also validate the caregivers’ expressed anxiety about the future care and provision for individuals with ID, as reported in previous research. Parents of individuals with ID commonly experience higher rates of stress, depression and anxiety because of the lifelong support that their children require, often placing the responsibility on the families (McConnell & Savage 2015; McKenzie, McConkey & Adnams 2013; Panicker & Ramesh 2019). The experiences described by the participants highlight the stigmatisation and discrimination faced by caregivers of individuals with ID when accessing healthcare, as supported by previous studies (Mkabile & Swartz 2020). Inadequate medical and care support often leads to referrals to healthcare professionals and social workers. However, there remains a shortage of appropriately qualified healthcare professionals in the field of ID (Coetzee et al. 2019).

The findings of this study highlight the profound psychosocial impact that caregiving for a person with ID has on caregivers. The shared experiences of family conflict among informal caregivers because of their caregiving responsibilities align with existing literature, which consistently reports a lack of social support among caregivers of individuals with IDs (Dada, Bastable & Halder 2020; Ugwuanyi et al. 2022). The evidence further reveals that a significant number of participants expressed their experiences of stigmatisation. This aligns with previous research, indicating that caregivers of individuals with ID face stigmatisation and discrimination, which create barriers when seeking access to healthcare (Mkabile & Swartz 2020; Scior et al. 2020).

The findings of this study highlight the pressing need for psychoeducation among both informal and formal caregivers of individuals with ID. Furthermore, supporting this finding, Petersen and Lund (2011) highlight the existence of significant deficiencies in community-based psychoeducational rehabilitation programmes in South Africa. They argue that the provision of community care is insufficient, as demonstrated by the high rates of hospital admissions and the reliance on police and prison services. Coetzee et al. (2019) support this perspective, stating that limited training opportunities are available for caregivers of individuals with ID in South Africa. Moreover, the literature reveals a lack of integration of ID-related programmes into academic and in-service training for practitioners in South Africa (Kleintjes et al. 2020; Smith Da Walt et al. 2018). This lack of integration hampers the development of competence in health, social care and education service delivery (Kleintjes et al. 2020). Collectively, these factors contribute to the need for improved psychoeducational resources and training programmes to enhance the skills and knowledge of caregivers in South Africa.

Furthermore, the study reveals that informal caregivers, often mothers, are predominantly responsible for the care of individuals with ID (Ezeonu et al. 2021; Mak & Cheung 2008; McKenzie 2016). Consequently, most caregivers are women, particularly those who are single, divorced or widowed (Ezeonu 2021; Lafferty et al. 2016; McKenzie 2016). These caregivers, mostly unpaid, assume their duties without any financial compensation. The Poverty Trends in South Africa report (2017) identifies vulnerable groups, including females, youth and individuals with no education, who are more susceptible to poverty. Coetzee (2016) asserts that caregivers experiencing poverty and hardship often struggle to allocate sufficient resources to meet the needs of individuals with ID. Moreover, the research findings indicate that caregivers often face challenges in pursuing their employment or are compelled to resign from formal employment because of their caregiving responsibilities (Gona et al. 2011). Capri et al. (2018) explain that the lack of educational programmes for individuals with ID in South Africa, coupled with the burden of caregiving and a lack of support, hinders informal caregivers from seeking employment opportunities. Nonetheless, informal caregiving remains the primary option for many individuals with ID in the Western Cape (McKenzie 2016).

Research studies conducted by McConnell and Savage (2015), Panicker and Ramesh (2019) and Steiner (2011) provide evidence that parents of children with ID experience elevated levels of stress and depression compared with parents of typically developing children. According to Steiner (2011), caregiver well-being, which encompasses reduced stress, symptoms of depression and negative affect, is closely linked to the behaviour of the individual with ID. However, McConnell and Savage (2015) argue that caregiver distress may precede and contribute to the behavioural difficulties exhibited by individuals with ID. They suggest that high levels of caregiver distress are associated with suboptimal caregiving practices, which in turn can lead to the emergence of behavioural problems in the individual with ID. Nonetheless, McConnell and Savage (2015) acknowledge that the relationship between caregiver distress and behavioural problems is often seen as a transactional process. The research results further correlate with literature, which indicates that psychoeducational programmes that have been documented to have catalysed improvements in caregiver well-being included educating caregivers regarding cognitive-behavioural therapeutic strategies to manage the challenging behaviour of the person with ID in their care (McConnell & Savage 2015; Steiner 2011).

The research results align with existing literature that highlights the effectiveness of psychoeducational programmes in improving caregiver well-being. These programmes focus on educating caregivers about cognitive-behavioural therapeutic strategies to manage the challenging behaviour of individuals with ID under their care (McConnell & Savage 2015; Steiner 2011). Moreover, in addition to teaching specific therapeutic techniques, the ability of caregivers to recognise and appreciate the positive attributes of the individuals they care for is crucial for positive outcomes (McConnell & Savage 2015; Steiner 2011). Identifying these positive characteristics in individuals with ID and nurturing the caregiver-person relationship can be particularly beneficial, considering that the stressors associated with the disability are long term. By adopting a strength-based cognitive approach, caregivers can develop a more positive perspective on the behaviour of individuals with IDs (Duan & Bu 2019; Steiner 2011).

A comprehensive psychoeducational programme for caregivers should also include a proactive plan for effectively managing challenging behaviour exhibited by individuals with IDs (Leoni et al. 2016). This is especially crucial for formally paid caregivers who often encounter distressing situations as part of their daily responsibilities in facilities catering to individuals with ID (Leoni et al. 2016). Frequent exposure to such incidents can potentially result in the development of maladaptive coping mechanisms, leading to detrimental effects on their mental health, psychological well-being, increased absenteeism and decreased productivity (Leoni et al. 2016). Consequently, the psychological distress experienced by formal caregivers can have significant financial implications for the organisation they work for (Leoni et al. 2016). Therefore, incorporating strategies to address the psychological well-being of formal caregivers is essential for promoting a healthy work environment and maintaining optimal caregiving standards.

The study demonstrated a significant strength in its implementation of a targeted psychoeducational intervention programme, which effectively alleviated caregiver distress. The respondents reported a reduction in feelings of social isolation, hopelessness, depression, anxiety and low self-esteem, all of which contributed to their sense of burden. These findings align with previous research conducted by Smith Da Walt et al. (2018) and support the effectiveness of such interventions in improving caregiver well-being.

The study was grounded in two theoretical frameworks: the biopsychosocial model and the strength-based approach. The biopsychosocial model provided a lens through which to understand the interplay between biological, psychological and social factors in caregiver well-being. This model recognised the significance of physical well-being, emotional well-being and the presence of a support network in shaping the overall well-being of caregivers. It highlighted the interconnectedness of these three domains and their influence on the caregiver’s experiences. The strength-based approach, on the other hand, framed the caregiving of individuals with ID within a framework of core strength principles. These principles encompassed social justice, transparency, empowerment, collaborative partnership, resilience and strength building. This approach emphasised the importance of recognising and harnessing the strengths and resources of caregivers, rather than solely focusing on deficits or challenges. It aimed to promote a positive and empowering caregiving experience by fostering collaboration, resilience and the enhancement of existing strengths. By integrating the biopsychosocial model and the strength-based approach, the study sought to provide a comprehensive understanding of caregiver well-being and highlight the potential for positive outcomes and growth within the caregiving process.

### Limitations

It is important to acknowledge that language barriers may have hindered the study, as the questionnaires were in English, which was not the first language for most of the respondents. This could have influenced how the questions were understood and answered, potentially impacting the research outcomes. Furthermore, the programme was designed as a one day training intervention, which may not have been sufficient considering the complexity of ID. Caregivers may require more extensive training than what was provided. Another limitation of the study was the lack of participation from individuals with ID. It is important to respect their unique perspectives and insights regarding their care needs and desired support from caregivers. Including their views would have added significant value to the psychoeducational programme. Additionally, the study relied on participants’ subjective evaluations and perceptions of their knowledge, rather than objective assessments. This introduces the possibility of bias or inaccurate self-assessments. Lastly, a notable limitation of the study was the use of a training manual developed based on interviews with informal caregivers, while the preliminary intervention involved formal caregivers. Formal caregivers may not have the same lived experiences as informal caregivers, potentially impacting their responses to the questionnaires.

## Conclusion

The findings of this study highlight the importance and positive impact of the psychoeducational programme in reducing caregiver distress and improving their psychological well-being. By addressing the existing gap in the literature, this study aimed to contribute significantly to the well-being of individuals with IDs by deepening the understanding and knowledge of their condition and enhancing the skills of their caregivers, including parents, siblings and neighbours.

Furthermore, the study also has broader implications for organisations that provide residential care to individuals with IDs. It offers valuable insights into the experiences and challenges faced by their employed caregivers who are entrusted with the care of these individuals. This information can empower management to re-evaluate the existing support structures in place and determine if they adequately meet the needs of formal caregiver employees. Additionally, it equips management with the necessary tools to enhance the skills and capabilities of their caregivers, ultimately improving the overall quality of care provided.

### Recommendations

It is crucial to recognise that caregivers of individuals with IDs have unique and varied needs. Therefore, it is imperative to involve and consult caregivers during the development of any caregiver support programme. The programme should be flexible and adaptable, allowing for customisation to address the evolving and diverse cultural needs of the caregivers. This approach also necessitates seeking input from caregivers when conducting evaluations of health and social services to ensure that their perspectives and experiences are considered.

Furthermore, it is essential to include caregivers and their families in the distribution of resources. These resources should be appropriately tailored and allocated to meet the specific needs of both the individual with ID and their caregiver household. By recognising the integral role that caregivers play in the lives of individuals with IDs, it becomes evident that supporting them through resource allocation is vital for enhancing the overall well-being and quality of care provided.

The next, critical, step is to refine the psychoeducational programme and continue educating caregivers on the most effective strategies for resilient caregiving. The findings from this study underscore the importance of fostering collaboration between social and healthcare service providers and caregivers to enhance the caregivers’ ability to provide care. It is essential to further explore the long-term impact of the implemented intervention programme on caregivers, individuals with ID, their families and the broader communities. Future research, particularly in the form of a longitudinal study, is necessary to determine whether the intervention programme has a sustained effect on all stakeholders involved.

Future research should prioritise gaining a comprehensive understanding of the phenomenon that places females in the role of caregivers for individuals with ID. This understanding can inform the development of policies, procedures and resource allocation strategies to address the specific needs of caregivers. Expanding the availability of the psychoeducational programme to a wider audience is recommended to alleviate caregiver distress on a broader scale. Building upon the findings of this study, it is recommended to further investigate the needs of caregivers in terms of resource distribution. This could involve exploring options such as providing financial grants to caregivers of individuals with ID, in addition to focusing on resources exclusively for individuals with IDs. Furthermore, future research should examine the relationship between social support structures and caregiver distress, with a focus on developing appropriate social support networks and interventions to alleviate the distress experienced by caregivers. Additionally, it is suggested that future research explores the development of early intervention programmes specifically tailored for caregivers whose child has been newly diagnosed with an ID. These programmes could encompass individual acceptance counselling, social support group interventions and psychoeducational training to equip caregivers with the necessary skills and knowledge to navigate the challenges associated with the diagnosis.
